# Treatment Strategies for Mucocutaneous Leishmaniasis

**DOI:** 10.4103/0974-777X.62879

**Published:** 2010

**Authors:** Emilio Palumbo

**Affiliations:** *Department of Pediatric, Hospital of Sondrio, Italy*

**Keywords:** Cutaneous leishmaniasis, Paromomycin, Pentavalent antimonials, Treatment

## Abstract

Mucocutaneous is an infection caused by a single celled parasite transmitted by sand fly bites. There are about 20 species of *Leishmania* that may cause mucocutaneous leishmaniasis. Some Leishmania species are closely linked to humans and are therefore found in cities (*L. tropica*) whereas some others are more traditionally associated with animal species and therefore considered zoonoses (*L. major*). The evidence for optimal treatment of mucocutaneous leishmaniasis is patchy. Although the cutaneous form of the disease is often self-limiting, it does result in significant scarring and can spread to more invasive, mucocutaneous disease. Therefore, treatment may be considered to prevent these complications. Drugs for systemic and topical treatment are presented and discussed with regard to their application, use and adverse effects.

## INTRODUCTION

The distribution of mucocutaneous leishmaniasis is very tightly linked to geography and villages even 15 miles apart can have very different rates of cutaneous leishmaniasis. There are about 20 species of *Leishmania* that may cause these diseases. Some Leishmania species are closely linked to humans and are therefore found in cities (*L. tropica*) some others are more traditionally associated with animal species and therefore considered zoonoses (*L. major*). Some species that are traditionally considered zoonotic (e.g. *L. panamensis*) becoming primarily human diseases of leishmania are transmitted to human skin by the bite of a sand fly. Leishmania invades human macrophages and replicates intracellularly. A raised, red lesion develops at the site of the bite (often weeks or sometimes years afterwards). The lesion then ulcerates and may become secondarily infected with bacteria. In many species (for example, *L. major*) the lesion often spontaneously heals with atrophic scarring. In some species (for example, *L. viannia braziliensis*) the lesion may spontaneously heal with scarring, but re-appear elsewhere as destructive mucocutaneous lesions. Lesions of other leishmania species may spontaneously heal and then re-appear as satellite lesions around the site of the original lesion, or along the route of lymphatic drainage. Some species tend to cause cutaneous and mucocutaneous leishmaniasis (*L. major* and *L. tropica*), whereas some species tend to cause visceral leishmaniasis (*L. infantum* and *L donovani.*[1–3] Diagnosis is based on the characteristic appearance of non-healing raised, scaling lesions that may ulcerate and become secondarily infected with organisms such as *Staphylococcus aureus*, in someone who has returned from an endemic area. The gold standard for diagnosis is PCR.[4] The evidence for optimal treatment of muco-cutaneous leishmaniasis is patchy. Although the cutaneous form of the disease is often self-limiting, it does result in significant scarring and can spread to more invasive, mucocutaneous disease. Therefore, treatment may be considered to prevent these complications.[5]

## TREATMENT STRATEGIES

Drugs for systemic and topical treatment are presented and discussed with regard to their application, use and adverse effects.

### Systemic treatment

The pentavalent antimonials are considered the gold standard for treatment of leishmaniasis. Two preparations are currently available abroad: sodium stibogluconate (Pentostam, GlaxoSmithKline) for intravenous and intramuscular administration and meglumine antimoniate (Glucantime, Specia Rhone Poulenc) for intramuscular administration. The biochemical basis for their effectiveness is unknown, but may involve inhibition of ATP synthesis. The drugs exist only in parenteral forms. The dosage is usually given in Sb equivalents (mg/kg/day). The pentavalent antimonials are far from ideal drugs because of their difficult administration and toxicity.[6,7] The adverse effects of the drug can also be significant and include pancreatitis, hepatitis, marrow suppression and changes to the electrocardiograph (QT prolongation). Other common adverse effects include myalgias, fatigue, headache, rash and nausea. In most instances, these events resolve when therapy is discontinued, and, therefore, weekly monitoring is recommended to rapidly detect toxicities and address them as they occur. Treatment schedules and dosages have been debated and changed several times. In the 1980s, the dosage was increased from 10 to 20 mg/kg/day, with an upper limit of 850 mg/day (equivalent to two ampoules of meglumine antimonate). The upper limit of 850 mg Sb/day was abandoned in the early 1990s, since studies indicated a reduced efficacy of lower doses and no higher toxicity was found with the higher doses of the drug.[8] Pentamidine, an aromatic diamidine, is toxic for a number of protozoa and fungi including *Leishmania, Pneumocystis carinii* and African trypanosomes. The mechanism of action has not been established. Pentamidine is used as an alternative to the pentavalent antimonials, and is the first line treatment for cutaneous leishmaniasis in French Guyana, where *L. guyanensis* is responsible for >90% of the cases.[9,10] A study in Colombia (with *L. panamensis, L. brasiliensis* and *L. mexicana*) found that a short-course, low-dose regimen of pentamidine isethionate had a similar cure rate (96%) to that of meglumine antimonate (91%), with similar rates of side effects.[11] The short-course, low-dosage regimen of pentamidine in otherwise healthy patients with cutaneous leishmaniasis was better tolerated than the higher dosages applied for *Pneumocystis* treatment in HIV-positive patients: all adverse effects were reversible and no cases of new diabetes mellitus were found among >2200 patients observed with the low-dose regimen. However, the higher dosages needed for the treatment of mucosal leishmaniasis (>2000 mg) may cause diabetes mellitus.[12] Blood sugar and glycemia need to be checked before every injection, because reversible glucosuria and hyperglycemia have been described.[13] Other side-effects are: nephrotoxicity (asymptomatic azotemia to renal failure), hypotension and arrhythmias, nausea, vomiting and diarrhea, leucopenia, anemia and thrombocytopenia, cough and bronchospasm, acute hepatitis and neurological alterations as neuralgia, confusion and hallucinations. The imidazoles and the structurally related triazoles were introduced as antifungal drugs, but also have an antileishmanial activity. They have the advantage of oral administration and few adverse effects, but are only effective against some species (see species-specific treatment below). Fluconazole was studied in a randomized, double-blind, placebo-controlled trial in Iran.[14] It was well tolerated and showed promising results in *Leishmania major* leishmaniasis. In a recent study a total of 106 patients were assigned to receive fluconazole, and 103 patients were assigned to receive placebo. Follow-up data were available for 80 and 65 patients, respectively. At the three-month follow-up, healing of lesions was complete for 63 of the 80 patients in the fluconazole group (79%) and 22 of the 65 patients in the placebo group (34%). According to an intention-to-treat analysis, the rates of healing were 59 and 22%, respectively. Sodium stibogluconate was offered to 11 patients in the fluconazole group who returned for follow-up and 33 of those in the placebo group (51%) in whom oral treatment was judged to have failed. Side effects were mild and similar in both groups. This study evidences as a six-week course of oral fluconazole is a safe and useful treatment for mucocutaneous leishmaniasis caused by *L. major.* Nausea, vomiting, diarrhea, stomach upset/pain, headache, dizziness, or hair loss may occur during treatment with fluconazole. This drug may rarely cause serious (possibly fatal) liver disease. A serious allergic reaction to this drug is unlikely, but seeks immediate medical attention if it occurs. Symptoms of a serious allergic reaction include: rash, itching, swelling, severe dizziness, trouble breathing. Miltefosine, a recent phosphocholine analogue, showed high *in vitro* activity against leishmania. Using doses of 133-150 mg/day for three to four weeks, the per protocol cure rate (no parasites after therapy, complete re-epithelialization after three months) was 94%. However, a longer follow-up is needed to evaluate the relapse rate. The most common side-effects were motion sickness, gastrointestinal complaints, headache and raised liver enzymes.[15] It has exhibited teratogenicity and should not be administered to pregnant women. The antifungal agent amphotericin B desoxycholate is active against *Leishmania* species, but has the disadvantage of a high incidence of adverse reactions (hyperpyrexia, severe malaise, hypotension, thrombophlebitis, azotaemia, renal tubular damage, hypokalemia, anemia and hepatitis). Based on currently available data, liposomal amphotericin B has been insufficiently studied with regard to formulation and dosage to assess its efficacy in mucocutaneous leishmaniasis. Allopurinol, an analogue of hypoxanthine, is generally not effective in the absence of pentavalent antimony.[16] Two azole antifungal agents, itraconazole and ketoconazole, have been used to treat mucocutaneous disease. Ketoconazole, which has the broadest spectrum, is unfortunately very poorly tolerated. Therapy-limiting adverse events include gastrointestinal symptoms (nausea, vomiting) as well as hepatotoxicity. Itraconazole, although better tolerated than ketoconazole, is associated with more treatment failures than the other azole compound.

## LOCAL TREATMENT

Topical therapies to treat cutaneous leishmaniasis include both pharmacologic and non-drug modalities. Non-drug therapy including cauterization, surgical excision, cryotherapy, but the simplest of these treatments is the local application of heat. As *Leishmania* species are heat labile, this topical therapy has been proven effective in placebo-controlled studies. There is an FDA-approved device (that treats cutaneous disease. This radio-frequency heat generator can be used either as a single treatment or as part of a multiple treatment course. Cryotherapy is the most common and it is performed by repeated topical applications of liquid nitrogen with a cotton-tipped applicator or a cotton swab with moderate pressure to the lesion, up to 2 mm outside the lesion margin. The freezing time per application is 15-20 second. The procedure is repeated two or three times at short intervals, resulting in a total time of 30-120 second. Adequate application is reflected in the whitening of the skin at 2-3 mm outside the margins of the lesion. In an non-randomized study from Turkey, the cure rates (complete healing and disappearance of all clinical features) after one or two sessions of cryotherapy were 77% (46/60) after one month and 73% (44/60) after three months, compared with 85% after 1 and 3 months following intralesional sodium stibogluconate.[17] Paromomycin, an aminoglycoside antibiotic (identical to aminosidine) has been used systemically against both visceral and cutaneous leishmaniasis. As an ointment for topical use, it has been tested in different formulations, either with methylbenzethonium in white soft paraffin, or with urea and white soft paraffin. The combination of paromomycin with methylbenzethonium appears to be more effective than the combination with urea, but it causes more local inflammatory reactions.[18] With New World leishmaniasis, however, experts are hesitant to treat cutaneous lesions topically because of the risk of mucosal disease, although this ointment was reported to have much better cure rates than placebo in two studies.[19,20] Local infiltration with pentavalent antimonial is an other therapeutic option. Local infiltration of lesions with pentavalent antimony produces the maximum concentration in the lesions and has few systemic side effects, but does not reach metastatic infections. The basic aim is to fill the infected part of the dermis with pentavalent antimony. This means carefully infiltrating the area around the lesion, including the base of the lesion. A fine gauge (25G) needle is used to inject the drug under pressure as the needle advances. Injection into the dermis is difficult, as the tissue space is small. The drug must not be injected into the subcutaneous tissue, where it is rapidly absorbed and does not reach the site of infection. In an Iranian study 96 patients with clinical and parasitological diagnosis of cutaneous leishmaniasis were recruited to a comparative randomized clinical trial to evaluate the efficacy of topical paromomycin vs. weekly intralesional injections of meglumine antimoniate. The patients were randomly divided into two treatment groups: one group was treated with topical paromomycin ointment and the other with intralesional meglumine antimoniate. Treatment was continued in both groups until complete recovery occurred (defined as healing in less than two months with no residual scar or relapse for up to one year post-treatment). Treatment failure was defined as an increase in the number and size of pre-existing lesions or untoward side-effects. The maximum treatment period was three months. The patients were followed up for one year. The results showed that intralesional meglumine antimoniate led to 41.7% complete recovery. However, topical paromomycin gave a lower cure rate of 16.6%. Treatment failure was observed in 39.7% of the group receiving intralesional meglumine and in 72.9% of those on topical paromomycin. This study indicates that intralesional meglumine antimoniate is superior to topical paromomycin in the treatment of cutaneous leishmaniasis.[21] Other topical creams that produce nitric oxide may also be beneficial for cutaneous leishmaniasis. These include SNAP (S-nitroso-N-acetylpenicillamine), ascorbic acid, salicylic acid and nitrite creams. Studies with each of these agents have had variable results. It does appear that the vehicle plays an important role in the effectiveness of topical treatments. Further studies are needed to determine their exact role in therapy.

## CONCLUSION

The pentavalent antimonials are considered the gold standard for treatment of leishmaniasis but the adverse effects of the drug can also be significant and include pancreatitis, hepatitis, marrow suppression and changes to the electrocardiograph. Also pentamidine is efficient but with many side effects as diabetes mellitus, nephrotoxicity, and anemia. The antifungal agent amphotericin B desoxycholate is active against *Leishmania* species, but has the disadvantage of a high incidence of adverse reactions. Itraconazolo and ketoconazole are generally well tolerated, but your use is limited by the fact that they demonstrate a slower activity against a limited number of strains. Flucanozole and miltefosine are generally well tolerated but other studies are needed to evaluate your efficacy. In consideration of toxicities of many drugs the selection of the therapeutic agent depends on the potential benefit of treatment versus the significant toxicities of many of these therapies.
